# Comparative Efficacy of Intrathecal Hyperbaric Levobupivacaine With Fentanyl Versus Hyperbaric Bupivacaine With Fentanyl in Elective Cesarean Section

**DOI:** 10.7759/cureus.70476

**Published:** 2024-09-29

**Authors:** Sinchana A S, Vijaykumar TK, Santosh Alalamath

**Affiliations:** 1 Anesthesiology, Shri B M Patil Medical College Hospital and Research Centre, Bijapur Liberal District Educational (BLDE) University, Vijayapura, IND

**Keywords:** bupivacaine, caesarean sections, fentanyl, levobupivacaine, local anesthetics, spinal anesthesia

## Abstract

Background

In parturient posted for elective cesarean sections, a widely used local anesthetic in the subarachnoid blockade is racemic hyperbaric bupivacaine. Hyperbaric levobupivacaine, the pure S (-) isomer of bupivacaine, was introduced and has since become more widely used in India because of its short duration of motor blockade and reduced risks of neurotoxicity and cardiotoxicity. This study aimed to compare the characteristics of sensory and motor blockade and side effects of equivalent doses of hyperbaric bupivacaine and levobupivacaine with opioid additive fentanyl in order to extend the duration of analgesia during elective cesarean births.

Methodology

A total of 130 women classified as American Society of Anesthesiologists (ASA) class I and II who are undergoing elective cesarean sections were enrolled in this randomized, prospective, double-blind comparative study after providing written informed consent. They were randomly assigned to one of two groups: Group LF, which included 65 women, obtained 25 mcg (0.5 ml) of fentanyl and 10 mg (2 ml) of hyperbaric levobupivacaine, or Group BF, which included 65 women, received 25 mcg (0.5 ml) of fentanyl and 10 mg (2 ml) of hyperbaric bupivacaine. Pinprick, cold swab, and the Bromage scale are used to assess the characteristics of sensory and motor blockade; hemodynamic alterations and adverse effects are also observed.

Results

The levobupivacaine group, consisting of 65 participants aged between 21 and 29 years, exhibited fewer side effects and significantly shorter durations of sensory and motor block compared to the bupivacaine group. In both groups, hemodynamic stability is comparable. Levobupivacaine provides analgesia for a significantly shorter period compared to bupivacaine.

Conclusions

In cesarean procedures, intrathecal hyperbaric levobupivacaine and fentanyl can be effective alternatives to hyperbaric bupivacaine and fentanyl due to their ability to maintain hemodynamic stability while providing adequate sensory and motor blockade.

## Introduction

Considering its faster onset and efficient sensory and motor blockage, the subarachnoid block is an effective way of delivering anesthetic during a cesarean delivery as opposed to general anesthesia. Bupivacaine is the most often used anesthetic drug for cesarean sections. It is available as a racemic mixture of its enantiomers, dextrobupivacaine and levobupivacaine. The plain bupivacaine is hypobaric, and using it may cause variable block height, possibly leading to a high block or block failure. The solution is made hyperbaric by the addition of 8% glucose to obtain a more reliable block. Administering local anesthetics via the spinal route is favored in cesarean deliveries as it offers analgesia and anesthesia. The drug’s volume, concentration, and dosage all have an impact on the effects [1]. The most frequently used local anesthetic for spinal anesthesia in women having cesarean sections is racemic bupivacaine.

Hyperbaric solutions often cause cephalad spread, which blocks cardiothoracic fibers and causes sudden bradycardia, hypotension, and cardiac arrest. Bupivacaine exhibits greater plasma protein affinity, binds more firmly to the receptor, and is removed extremely slowly from the isolated nerves, resulting in a prolonged duration of the block. Bupivacaine crosses the blood-brain barrier, and systemic absorption or direct intravascular injection can lead to CNS toxicity. Cardiotoxicity is primarily due to direct blockade of cardiac Na+ channels and inhibition of the autonomic nervous system. Bradycardia, heart block, and hypotension may lead to cardiac arrest. High protein binding of bupivacaine complicates resuscitation efforts, especially in cases of pregnancy.

Levobupivacaine is the pure S isomer of bupivacaine and can be used in place of bupivacaine due to its lower cardiotoxicity and neurotoxicity [2]. The S (−) isomer exhibits weaker potassium channel-blocking potency, reducing the likelihood of QTc interval prolongation. Stereoselective binding to sodium and potassium channels decreases the inhibitory effects, thereby lowering the overall toxicity potential compared to bupivacaine. It also provides extended advantages because of its more pronounced affinity for sensory fibers than motor fibers and provides predictable spread after spinal anesthesia [3].

Incorporating low doses of opioids [4] with local anesthetic agents during subarachnoid blockade reduces the side effects associated with local anesthetics and prolongs their duration of action. It provides intra- and postoperative analgesia. Fentanyl can be added as it increases the duration of action [5] and also helps by reducing the dose of local anesthetic, thus reducing its side effects.

Therefore, the goal of this analysis is to assess the levels of the sensory and motor blockade with the adverse effects that arise from intrathecal fentanyl mixed with equivalent dosages of hyperbaric solutions of bupivacaine and levobupivacaine during elective cesarean sections.

## Materials and methods

This randomized prospective double-blinded comparative study was conducted for one year from March 2023 to March 2024 after approval from the institutional ethical committee. The study is registered with the Clinical Trials Registry - India (CTRI) under registration number CTRI/2023/03/050728. Double blinding was ensured by hiding the group allocation, the name of the drug, and the person who recorded the data.

Data was analyzed using G*Power version 3.1.9.4 software for sample size calculation. The weight (kg) of pregnant women posted for elective cesarean section (mean = 75, SD = 19) and control (mean = 82, SD = 12). This study requires a total sample size of 130 (65 for each group), assuming equal group sizes. In order to achieve a power of 80% for detecting a difference in means, t-tests (means: the difference between two independent means (two groups)) were calculated.

One hundred thirty randomly selected participants who belong to the American Society of Anesthesiologists (ASA) physical status I and II scheduled for elective cesarean delivery at a gestational age greater than 37 weeks participated in this study. A pregnant woman who weighed more than 100 kg and was shorter than 150 cm, woman with fetal abnormality, placenta previa, or abruption placenta, height greater than 175 cm, history of prior systemic disorders, medication history (apart from vitamin, calcium, and iron), and contraindications to spinal anesthesia were excluded from the study.

During preanesthetic evaluation, a detailed history and general and systemic examination were carried out the previous day. A history of any significant medical illness was elicited, and a medication history was taken. The airway, respiratory system, and cardiovascular system were assessed. Written informed consent was obtained. Routine investigations like complete hemogram, bleeding and clotting time, random blood sugar, and serology (hepatitis B and HIV) were performed.

Before surgery, participants were evaluated, and on the day of the procedure, nil per oral condition was verified. A 20-gauge IV cannula provides intravenous access, and Ringer’s lactate started at 15 ml/kg/hour. Following the transition to the standard surgical table, monitoring instruments such as pulse oximeters, noninvasive blood pressure, and ECG were connected, and their baseline values were collected. Parturients were put in the left lateral decubitus position, and painting and drapery were completed while adhering to aseptic protocols. Subcutaneous infiltration was carried out at the L3-L4 junction using 1-2 ml of 2% lignocaine. A 26G Quincke's spinal needle was used for lumbar puncture, and the subarachnoid space was located.

Parturients were split into two groups at random. Group Bupivacaine with fentanyl (Group BF), consisting of 65 patients, was given 25 mcg (0.5 ml) of fentanyl and 10 mg 0.5% (2 ml) of hyperbaric bupivacaine. Sixty-five patients in Group Levobupivacaine with fentanyl (Group LF) were administered 10 mg 0.5% (2 ml) of hyperbaric levobupivacaine with 0.5 ml or 25 mcg of fentanyl. Drugs were given within 10 seconds, intrathecally. Parturients were positioned supine, and oxygen was administered at 4 L/min.

Using the Bromage scale for the motor level assessment and a pinprick and cold swab for the sensory level assessment, the sensory blockade level was confirmed bilaterally in the midclavicular line. Once the level reaches T4 to T6, the surgeon is allowed to operate. The duration of spinal anesthesia administration was noted, then pulse rate, systolic blood pressure (SBP), diastolic blood pressure (DBP), mean arterial pressure, and saturation, which were measured every two minutes starting at time 0 for the first 60 minutes. After that, vitals were measured every 30 minutes until the procedure was completed and every hour for the next 24 hours after the surgery.

The beginning of the sensory block, its duration, the time taken for maximum block, and the time for the two block dermatomes to regress were all observed from time zero, which indicates intrathecal injection time. The onset of sensory block was considered when the amount of time passed between the study drug’s administration and the T10 dermatome level pain prick test’s disappearance. The time taken for the two-segment regression of the sensory block was defined as the time taken from the beginning of the onset of the block at T10 and its regression to the T12 dermatome level. The duration of the block was calculated from the point when the patient first experienced a sensory block and its regression to the S2 dermatome level.

The time interval from study drug injection to motor paralysis equal to a Bromage score of 3 was used to determine motor block onset. The modified Bromage scale was used to measure the degree of motor block. A patient with a Bromage of 0 is able to lift their leg against gravity and move their hip, knee, and ankle. Bromage 1 denotes the ability to flex the knee and ankle but not the ability to raise the leg against gravity. Bromage 2 shows a failure to extend the ankle flexion and the hips and knees. Bromage 3 denotes the inability to bend the ankle, knee, and joint but the ability to move the toes. Bromage 4 denotes total paralysis. The length of a motor block was measured from the time it began until complete motor function reverted to a Bromage score of 0. Episodes of bradycardia, hypotension, nausea, and vomiting were observed. Adverse effects like shivering, itching, and headaches were noted.

The data obtained was entered into a Microsoft Excel sheet (Microsoft Corporation, Redmond, WA, USA), and statistical analyses were performed using IBM SPSS Statistics for Windows, Version 20.0 (Released 2011; IBM Corp., Armonk, NY, USA). Results were presented as mean, SD, counts and percentages, and diagrams. In normally distributed continuous variables between the two groups, results will be compared using an independent sample t-test. In cases of variables that are not distributed normally, the Mann-Whitney U test was used. In categorical variables, results were compared using the chi-square test or Fisher’s exact test. p < 0.05 is considered statistically significant. All statistical analyses were performed in two-tailed.

## Results

**Figure 1:**
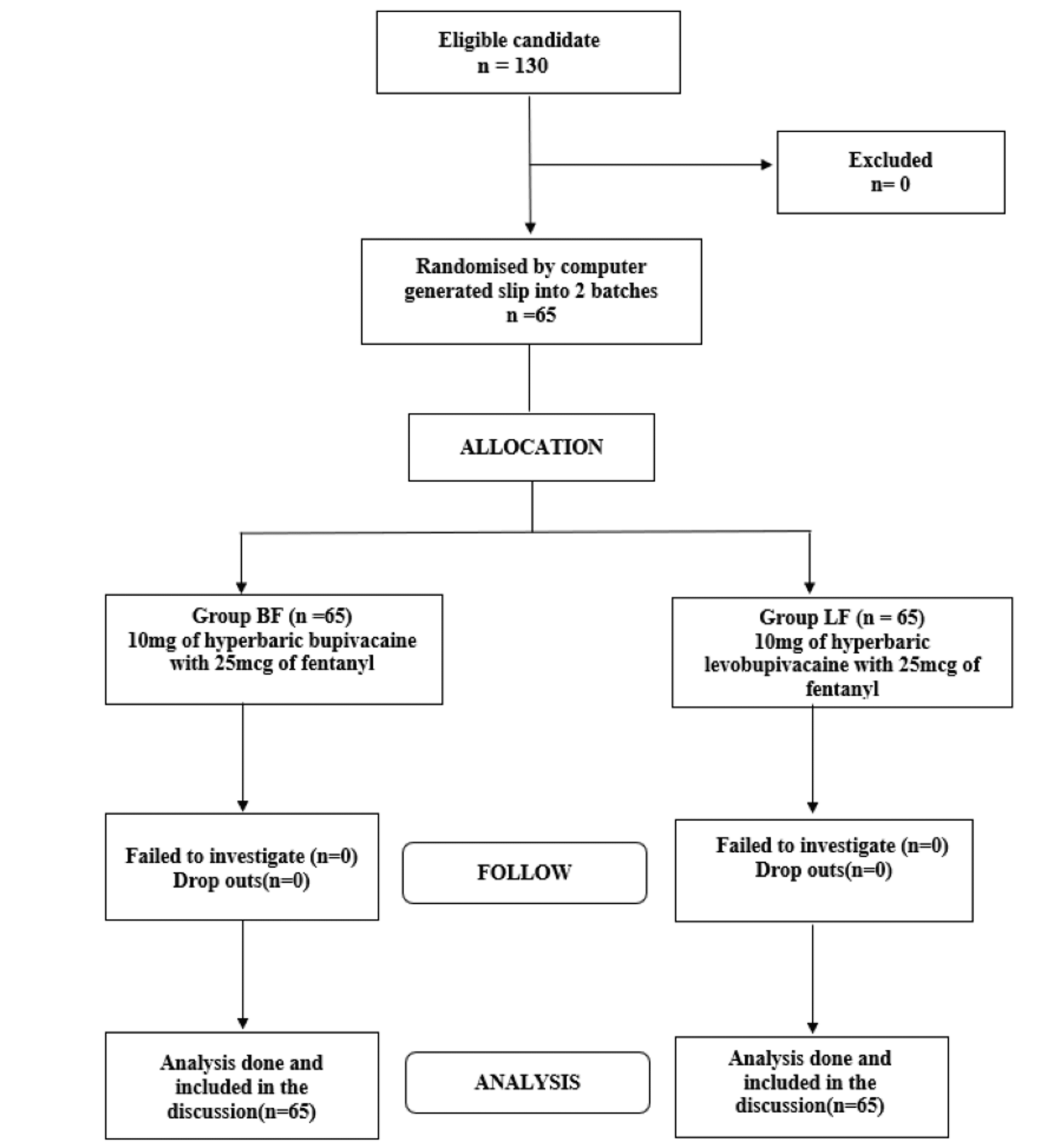
CONSORT flow diagram CONSORT, Consolidated Standards of Reporting Trials

In this study, 130 women were posted for elective cesarean, and 65 in each group were compared for anesthetic effectiveness. We have followed the Consolidated Standards of Reporting Trial (CONSORT) guidelines in our study (Figure 1).

The mean values of age, weight, and gestation week were compared between the two groups. Age in years and weight in kilograms were calculated using the Mann-Whitney U test. There was no significant statistical difference between age and weight characteristics in either group (significant value is p < 0.05). The mean gestation week was calculated between the two groups using the Mann-Whitney U test and showed significant differences with a p-value of 0.029 (Table 1).

**Table 1:** Comparison of demographic characteristics between two groups The demographic characters were calculated using the Mann-Whitney U test and are considered significant when the p-value is <0.05. Age and weight have no statistically significant difference between the two groups, but gestation week shows a significant difference with a p-value of 0.029.

Variable	Group BF	Group LF	p-value
Age	25.23 ± 4.63	26.14 ± 4.86	0.2
Weight	62.9 ± 7.85	62.7 ± 8.73	0.966
Gestation week	38.1 ± 1.54	37.5 ± 1.85	0.029

The mean duration of onset of sensory block, time to reach T10, time to reach maximum level, time of regression of 2 dermatomes, total duration of sensory block, onset of motor block, and total duration of analgesia were compared between the two groups. It was considered significant when the p-value was <0.05. Although the onset and peak level time of sensory and motor blockade were similar in both groups, the overall duration of sensory and motor blockade was significantly shorter with levobupivacaine than with bupivacaine (Table 2).

**Table 2:** Comparison of characteristics of anesthesia between two groups p < 0.05 is considered statistically significant.

Variables (in minutes)	Group BF	Group LF	p-value
Onset of sensory block	1.11 ± 0.6	1.51 ± 0.8	0.11
Time to reach T10	1.95 ± 1.25	2.30 ± 1.18	0.517
Time to reach T4	0.84 ± 2.89	0.46 ± 1.29	0.51
Time for regression of 2 dermatomes	177.25 ± 35.6	151.78 ± 35.0	0
Total duration of sensory block	194.6 ± 30.5	166.7 ± 31.9	0
Onset of motor block	1.45 ± 0.99	1.48 ± 0.66	0.125
Time to reach the maximum level	2.74 ± 1.82	2.83 ± 1.1	0.105
Duration of analgesia	196.35 ± 29.6	170.46 ± 34.4	0

**Figure 2:**
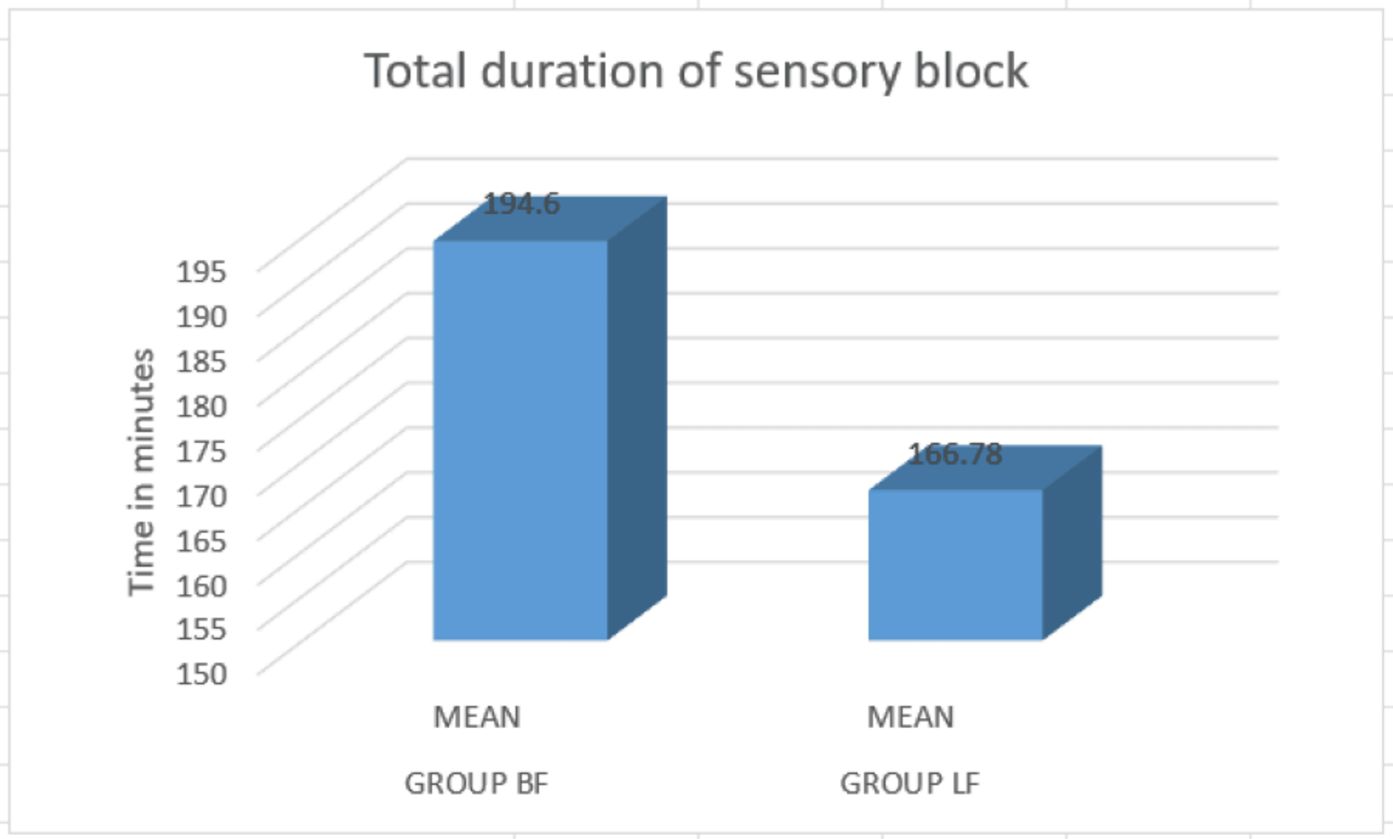
Comparison of duration of sensory block When comparing the mean duration of sensory block, the levobupivacaine and fentanyl group exhibited a shorter duration of 166.76 minutes, in contrast to the bupivacaine and fentanyl group, which had a mean duration of 194.6 minutes.

The levobupivacaine group showed a significantly shorter total duration of sensory block compared to the bupivacaine group. The average duration of sensory block was notably shorter in the levobupivacaine group (166.78 minutes) than in the bupivacaine group (194.6 minutes) (Figure 2).

**Figure 3:**
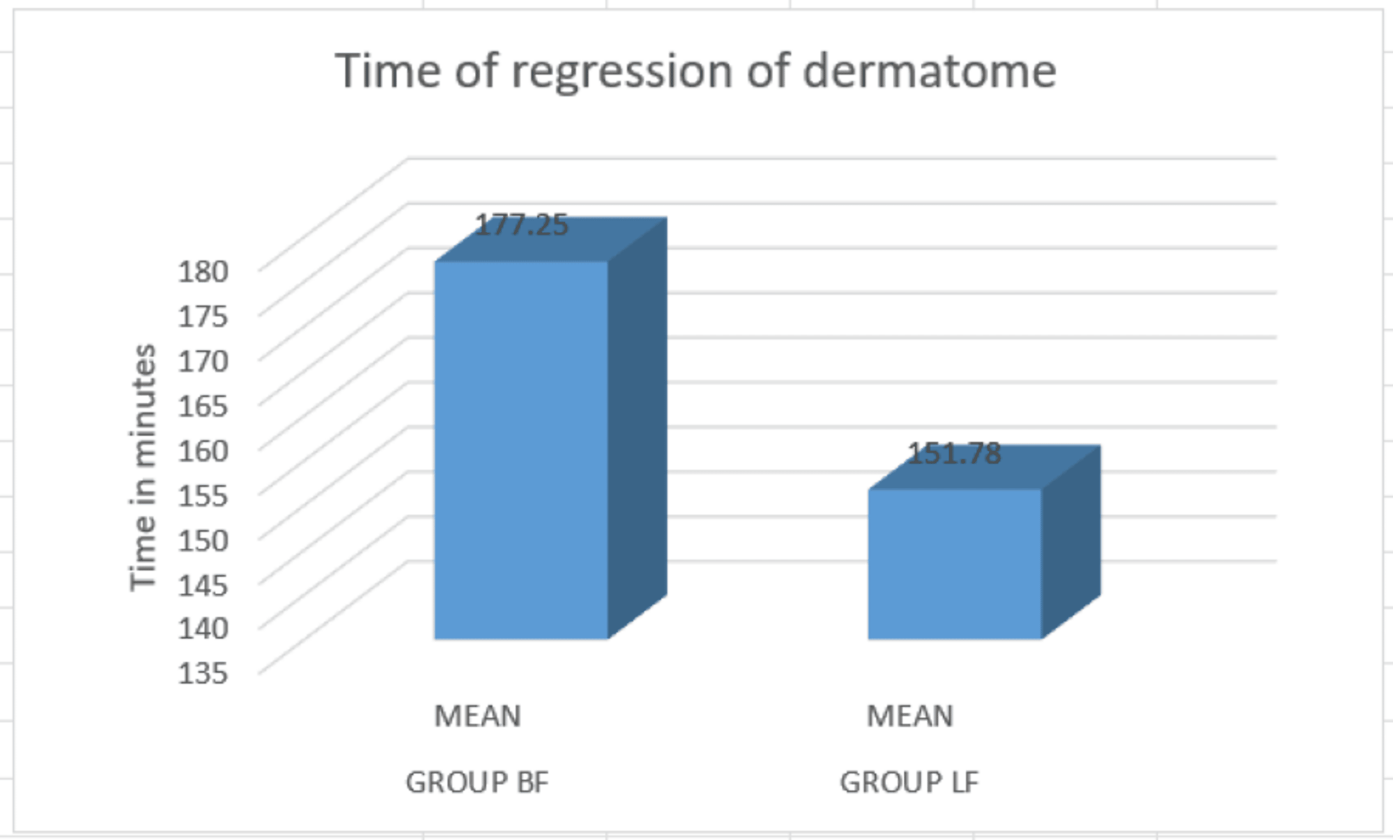
Comparison of regression of dermatome The regression of dermatome was shorter duration with levobupivacaine and fentanyl group 151.78 minutes compared to bupivacaine and fentanyl group 177.25 minutes.

There was a significant difference in the mean regression of dermatomes between the two groups. The mean regression of dermatome was higher in the bupivacaine group (177.25 minutes) compared to the levobupivacaine group (151.78 minutes) (Figure 3).

**Figure 4:**
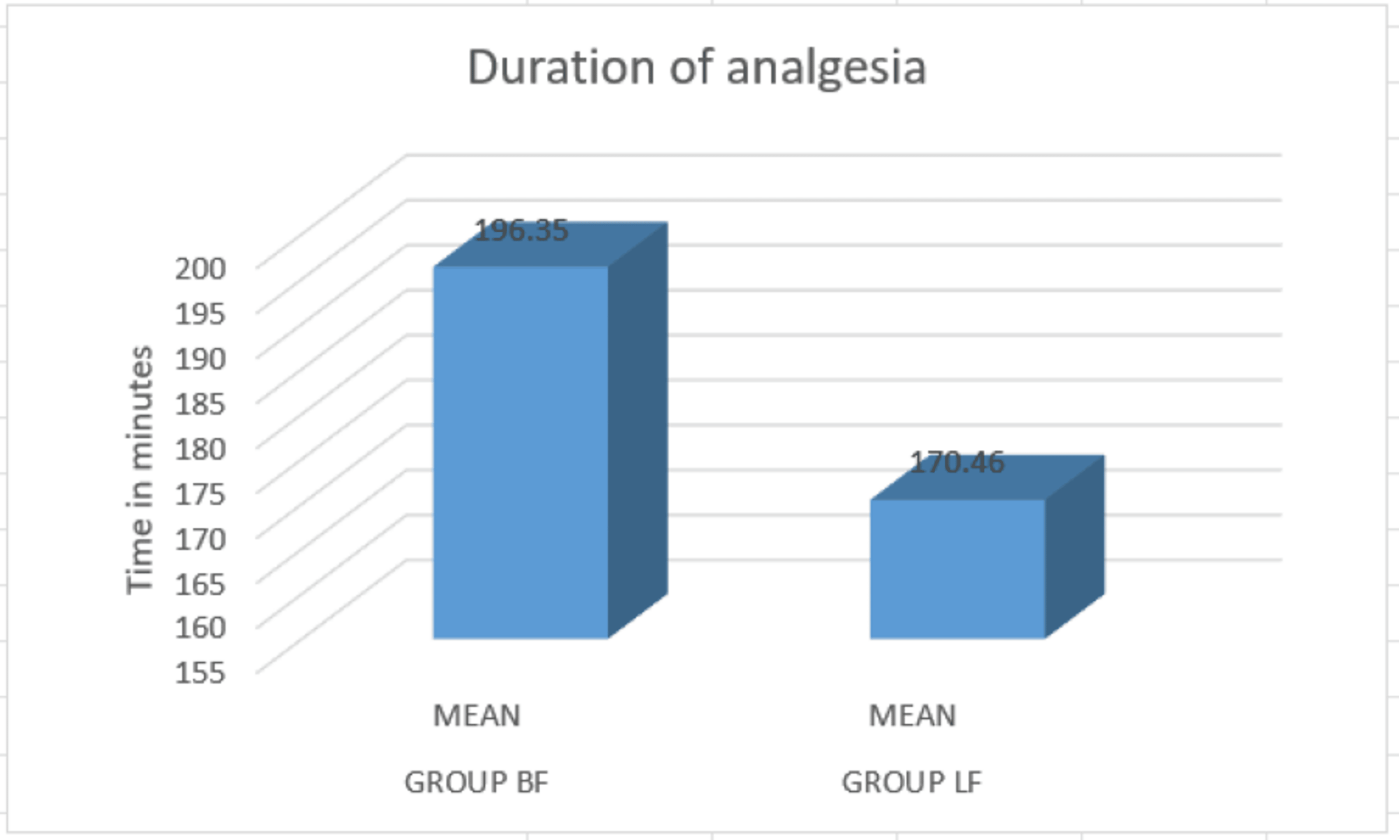
Comparison of total duration of analgesia The mean total duration of analgesia was compared, revealing that the levobupivacaine plus fentanyl group provided a shorter duration of 170.46 minutes compared to the bupivacaine plus fentanyl group, which lasted 196.35 minutes. This difference facilitates earlier ambulation.

Levobupivacaine provided a shorter total duration of analgesia (170 ± 34.4 minutes) compared to the bupivacaine group (196.35 ± 29.6 minutes) (Figure 4).

**Figure 5:**
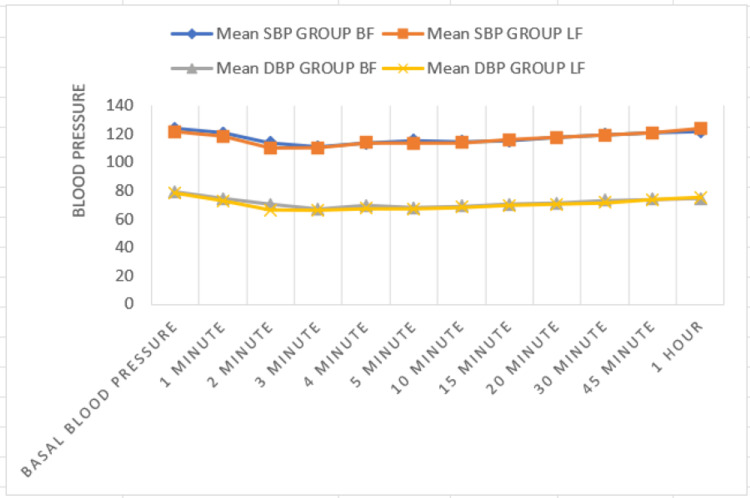
Intraoperative blood pressure recordings between Group BF and Group LF Significant variations were not observed when mean SBP and DBP were compared between levobupivacaine plus fentanyl group and bupivacaine plus fentanyl group. DBP, diastolic blood pressure; SBP, systolic blood pressure

In our study, drops in SBP and DBP were within acceptable limits, with the bupivacaine group showing a 43.1% decrease and the levobupivacaine group showing a 30.8% decrease. While the occurrence of hypotension was similar in both groups, the combination of levobupivacaine and fentanyl provided the same hemodynamic stability as the bupivacaine and fentanyl groups. Hypotension is considered when SBP is below 90 mmHg (Figure 5).

The occurrence of bradycardia was similar in both groups. Itching was more prevalent in the bupivacaine group (20%) than in the levobupivacaine group (4.6%). Bupivacaine group experienced more shivering (36.1%) compared to levobupivacaine group (13.8) (Table 3).

**Table 3:** Side effects in Group BF and Group LF Side effects associated with both groups were compared using the chi-square test. p < 0.05 is considered statistically significant.

Side effects	Group BF		Group LF		p-value
	N	%	N	%	
Hypotension	28	43.1	20	30.8	0.146
Bradycardia	11	16.9	11	16.9	1
Nausea and vomiting	23	35.4	11	16.9	0.017
Headache	10	15.4	6	9.2	0.286
Itching	13	20	3	4.6	0.008
Shivering	24	36.9	9	13.8	0.003

## Discussion

This study compares the effects of hyperbaric bupivacaine 10 mg and hyperbaric levobupivacaine 10 mg, both combined with 25 mcg of fentanyl, in elective cesarean sections. Both drug combinations produce similar quality in the onset of sensory and motor blockade, with comparable maternal hemodynamic outcomes. However, less duration of sensory and motor blockade is witnessed with levobupivacaine [6] when compared to bupivacaine, which provides a longer-lasting analgesic effect.

The efficacy of local anesthetics in the neuraxial blockade is empowered by the addition of opioids, as they increase the duration of analgesia and maintain hemodynamic stability through dose reduction of the local anesthetics [7]. Postoperative analgesia increases with higher doses of bupivacaine [8]. Adding fentanyl [9] to bupivacaine enhances postoperative analgesia due to its synergistic effect, though it also prolongs motor recovery.

Bogra et al. found that intrathecal fentanyl in elective cesarean sections creates a synergistic effect due to its fast onset and short duration, with fewer respiratory issues. The study was conducted in 120 patients; the first group of 60 participants was further subdivided into three subgroups receiving 8, 10, or 12.5 mg of 0.5% hyperbaric intrathecal bupivacaine, respectively. The second group, also of 60 participants, was again subdivided into three subgroups receiving 8, 10, or 12.5 mg of 0.5% hyperbaric intrathecal bupivacaine mixed with 12.5 μg of intrathecal fentanyl. The onset of sensory block occurred faster with increasing bupivacaine doses in bupivacaine-only groups and bupivacaine-fentanyl combination groups. The incidence of nausea and shivering reduces significantly, whereas postoperative pain relief and hemodynamics increase by adding fentanyl. Intraoperative sedation was not seen; however, bupivacaine and fentanyl group participants were drowsy but arousable. Side effects like pruritus and maternal respiratory depression are minimal with fentanyl [10].

Lee et al. compared the clinical efficacy, motor block, and hemodynamic effects of using 2.6 mL of 0.5% levobupivacaine alone (25 patients) and 2.3 mL of 0.5% levobupivacaine with fentanyl (15 μg) in 0.3 mL (25 patients) in a urological study and reported that using levobupivacaine 0.5% with fentanyl in urological surgery is as effective as using levobupivacaine alone. This shows that reducing the dose of local anesthetic levobupivacaine by adding fentanyl has no effects on hemodynamic stability, but levobupivacaine provides a shorter duration of motor block, which helps the patient in early ambulation and less duration of hospital stays [11].

Bidikar et al. compared levobupivacaine 0.5% 10 mg with a total volume of 2 ml or levobupivacaine 7.5 mg plus fentanyl 12.5 μg and found that the combination extended sensory block duration and delayed the need for additional analgesia without prolonging motor block. The hemodynamics were similar in both groups. The clinical significance of reduced duration of the motor block resulting from lower-dose levobupivacaine plus fentanyl would be early ambulation, which is similar to our study [12].

In a study conducted by Bremerich et al. [13], the authors compared hyperbaric bupivacaine 0.5% with hyperbaric levobupivacaine 0.5%, both combined with opioids: either fentanyl in doses of 10 or 20 mcg or sufentanil at 5 mcg. Sensory and motor block characteristics, as well as postoperative analgesic effects, were evaluated. Their outcome showed that for all opioids used, levobupivacaine caused shorter and less effective motor blockade than bupivacaine. Notably, no participants experienced pain during surgery. Additionally, the incorporation of sufentanil 5 mcg with either local anesthetic notably extended the duration of adequate analgesia more than the supplemental fentanyl doses of 10 or 20 mcg. This study shows that the addition of opioids provides postoperative analgesia without any motor block, aiding in early recovery.

A study by Abd-El Wahab et al. compared 2.5 ml of 0.5% levobupivacaine and 2.5 ml of 0.5% bupivacaine in 60 participants belonging to ASA grades 1 and 2 scheduled for elective cesarean sections, finding that both provide adequate surgical anesthesia. Levobupivacaine intrathecally offers a safer option due to lower incidences of cardiotoxicity and neurotoxicity. It has been found to be equally efficacious as bupivacaine, but with a superior pharmacokinetic profile. In agreement with our study, the onset of sensory and motor block, the time of maximum sensory and motor block level, and the time taken for regression of motor block were significantly shorter in the levobupivacaine group. As regards heart rate and mean arterial blood pressure, the study found that it was insignificant statistically when both groups were compared with each other. Hence, levobupivacaine is a safer alternative to bupivacaine [14].

However, hypotension is common during spinal anesthesia due to CSF displacement caused by pregnancy-related engorgement of epidural veins. Hypotension is the most common side effect seen with more than 50% of parturients after subarachnoid blockade due to swelling of epidural veins resulting from aortocaval compression [15], which causes displacement of CSF and results in more cephalad spread of local anesthetic and increasing the risk of hypotension. The cephalad spread of blockade beyond T4 produces bradycardia [16]. Fentanyl used as an additive most frequently causes itching [17].

Limitations

Our study’s main limitation lies in the fact it was confined to ASA physical status I and II participants undergoing elective cesarean sections, which limits the homogeneity of the population and limits the drug's generalizability. It may also be less applicable to a wider range of patients if individuals with comorbidities, weights over 100 kg, or heights over 175 cm are excluded. In future studies, analyzing the effectiveness of different adjuvants may provide insightful information to improve spinal anesthetic techniques for better clinical outcomes.

## Conclusions

Both hyperbaric levobupivacaine and hyperbaric bupivacaine effectively and rapidly provide surgical anesthesia for elective cesarean deliveries, making them reliable choices for this procedure. Combining levobupivacaine with fentanyl results in the same hemodynamic stability as hyperbaric bupivacaine and reduced side effects. This combination reduces risks and facilitates early mobility for mothers following the elective surgery. The combination of levobupivacaine with fentanyl is preferred for elective cesarean sections due to its minimized motor blockade, improved maternal comfort, and favorable safety profile.
